# Optimization of enzyme-assisted microwave extraction of *Zanthoxylum limonella* essential oil using response surface methodology

**DOI:** 10.1038/s41598-023-40142-4

**Published:** 2023-08-08

**Authors:** Sarunpron Khruengsai, Nittirat Promhom, Teerapong Sripahco, Piyanuch Siriwat, Patcharee Pripdeevech

**Affiliations:** 1 School of Science, Mae Fah Luang University, Chiang Rai, 57100 Thailand; 2 Center of Chemical Innovation for Sustainability (CIS), Mae Fah Luang University, Chiang Rai, 57100 Thailand

**Keywords:** Biotechnology, Developmental biology

## Abstract

*Zanthoxylum limonella* essential oil possesses potential antimicrobial activity and is of considerable interest as food flavouring and traditional herb. In this study, an enzymolysis-pretreatment-microwave-assisted extraction (EP-MAE) method was used to extract *Z. limonella* essential oil. The response surface methodology (RSM) with Plackett–Burman design (PBD) and Box-Behnken design (BBD) models were employed to optimize conditions in the EP-MAE method. Seven variables including water to plant ratio, enzyme amount, incubation temperature, incubation time, shaking speed, microwave time, and microwave power were selected to determine the optimal values for extracting *Z. limonella* essential oil. As the results, four variables including water to plant ratio, enzyme amount, microwave time and power were evaluated as significant variables affecting on yield and volatile compounds of *Z. limonella* essential oil from both PBD and BBD experiments. The optimum conditions of EP-MAE was obtained as follows: water to plant ratio (11.16 mL/g), enzyme amount (0.68%), microwave time (36.73 min), and power (1665 W). The *Z. limonella* essential oil composition and its yield from EP-MAE was compared to those extracted from MAE and hydrodistillation. The optimal extraction conditions in the EP-MAE method enhanced significantly higher essential oil yield (7.89 ± 0.08 mg/g) compared to those found by MAE (7.26 ± 0.04 mg/g) and hydrodistillation (7.04 ± 0.03 mg/g), respectively. Fifty-one volatile components were identified among these methods, with similar major compounds of limonene, β-pinene, and α-phellandrene, showing percentage ranging between 34.59–35.78%, 19.91–22.67%, 8.47–8.75%, respectively. However, an extremely higher content of compounds was detected using the EP-MAE method. This study demonstrates the significance of EP-MAE, which may be applied as a more potent extraction method for essential oils in aromatic plants compared to MAE and hydrodistillation.

## Introduction

Essential oils are viscous liquids containing complex components with aromatic odour. They have been broadly used in various applications in pharmaceuticals and natural therapies, as well as food industries since ancient time until now^1,2^. They have also been applied as antimicrobial and antioxidant agents for food security, replacing chemical and synthetic drugs^1,2^. Generally, essential oil is mainly extracted by hydrodistillation due to its ease of operation and low cost. However, this technique consumes both time and energy. In addition, this method often results in low yields and the production of degradation products, including thermosensitive components^3^. Due to the high demand of essential oils, there is a growing concern for improving yields. Development of novel cost-effective and environmentally friendly techniques are encouraged to extract essential oils. Microwave-assisted extraction (MAE) is a novel and efficient method for extracting essential oil significantly reducing extraction times and enhancing essential oil yield^4^.

MAE technique is widely known as green, and efficient technique for extracting essential oil without use of organic solvents^5,6^. The extraction of essential oils through the MAE technique is directly correlated to the microwave radiation's interaction with polar compounds and water, leading to elevated temperatures and pressures within the plant cells^6,7^. The essential oil disperses easily and rapidly from aromatic plant reducing time and energy consumption^7^. In this method, essential oil yield and content of volatile compounds are also enhanced. Moreover, enzymolysis pretreatment (EP) is known to improve essential oil yield from aromatic plants by interfacing with extraction techniques such as MAE according to high efficiency, low cost of enzyme concentration, simple method, and friendly environment^8,9^. Various hydrolytic enzymes are used in this approach to destroy the plant cell cytoderm and easy release endocellular components^8,9^. Therefore, using an alternative method of enzymolysis pretreatment combined with microwave irradiation has been interested and quickly increased for extraction of essential oils^8,10^.

*Zanthoxylum limonella* is a perennial tree belonging to the Rutaceae family. It is normally cultivated in Southeast Asia^11^. It is generally planted for the purpose of food flavouring and traditional herb for treatment of several diseases such as stomach, and tooth infections and respiratory diseases^12^. Utilization of *Z. limonella* is also detected as a source of strong antimicrobial agents which has been used as food additives with major components of limonene, phellandrene, and sabinene^11–13^. Hydrodistillation has been major technique for extracting *Z. limonella* essential oil. However, using new extraction technology instead of conventional hydrodistillation to enhance yield of *Z. limonella* essential oil is still limited. Thus, efficient method for with great ecological and economic benefits has been investigated to extract of essential oil from *Z. limonella* fruits^13^.

In this work, the EP-MAE was employed to extract *Z. limonella* essential oil and the extraction conditions were optimized using RSM with PBD and BBD models. Yield of *Z. limonella* essential oil extracted by MAE and hydrodistillation were also compared with those extracted by EP-MAE method. *Z. limonella* essential oil composition obtained by all methods was also analysed by gas chromatography-mass spectrometry (GC–MS).

## Results

### Screening of significant variables by PBD

**Figure 1:**
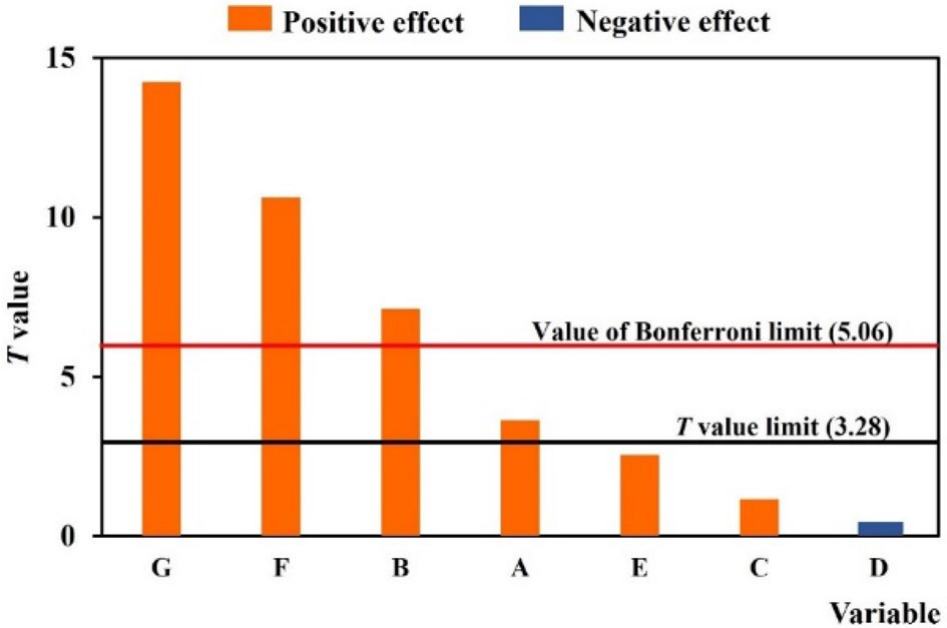
Pareto chart of the *Z. limonella* essential oil yield from EP-MAE from PBD model with variables including (**A**) water to plant ratio, (**B**) enzyme amount, (**C**) incubation temperature, (**D**) incubation time, (**E**) shaking speed, (**F**) microwave time, and (**G**) microwave power.

Seven variables (Table 1) including water to plant ratio, enzyme amount, incubation temperature, incubation time, shaking speed, microwave time, and microwave power were employed to optimize the suitable values for extracting *Z. limonella* essential oil. All selected variables affecting on yield of *Z. limonella* essential oil was evaluated by PBD model with twelve trial runs (Table 2). From PBD design, the regression equation was achieved which was able to predict the variables influenced essential oil yield. The model equation for the yield of *Z. limonella* oil can be achieved as Y = 1.40 + 0.05A + 0.92B + 0.01C-0.01D + 0.01E + 0.02F + 0.01G. Figure 1 shows the significance of each variable via a Pareto chart with *T* and *P* values. As the results, the sign—could reflect the negative effect whereas the sign + of *T* values indicated positive effect of variables on the extraction condition. The variables were higher than the *T* value limit (3.28) and the Bonferroni limit (5.06) were determined as significant variables in EP-MAE method. It was found that four variables including microwave time and power, enzyme amount, and water to plant ratio are considered as the significant variables on yield of *Z. limonella* essential oil in the EP-MAE method.

**Table 1:** The variables and levels used in PBD and BBD experiments.

Variable	low level (− 1)	Central level (0)	high level (+ 1)
A: water to plant ratio (mL/g)	8	10	12
B: enzyme amount (%)	0.4	0.6	0.8
C: incubation temperature (°C)	40	50	60
D: incubation time (h)	2	3	4
E: shaking speed (rpm)	200	400	600
F: microwave time (min)	20	30	40
G: microwave power (W)	1300	1500	1700

**Table 2:** PBD design results for eight variables in coded and real values.

Run	A	B	C	D	E	F	G	Yield (%)	
								Actual	Predicted
1	8	0.8	40	4	600	20	1700	6.54	6.54
2	8	0.4	40	2	200	20	1300	5.23	5.30
3	8	0.8	60	4	200	20	1300	5.72	5.73
4	8	0.4	60	2	600	40	1300	6.05	6.04
5	8	0.8	60	2	600	40	1700	7.12	7.14
6	8	0.4	40	4	200	40	1700	6.65	6.57
7	12	0.8	40	4	600	40	1300	6.48	6.52
8	12	0.4	60	4	600	20	1300	5.76	5.70
9	12	0.8	60	2	200	20	1700	6.72	6.69
10	12	0.4	60	4	200	40	1700	6.74	6.82
11	12	0.4	40	2	600	20	1700	6.37	6.38
12	12	0.8	40	2	200	40	1300	6.45	6.40

ANOVA									
Source	Sum of squares	Degree of freedom	Mean square	*F* value	*P *value	Inference			
model	3.08	7	0.44	54.62	0.0008	***			
residual	0.03	4	0.01						
Cor total	3.11	11							

Regression data									
Term	Effect	Coefficient	S.E.	F value	T value	*P* value	Inference		
A	0.20	0.05	0.01	15.14	3.63	0.0177	*		
B	0.37	0.92	0.12	51.43	7.11	0.0020	**		
C	0.07	0.01	0.01	1.57	1.15	0.2781	ns		
D	− 0.01	− 0.01	0.02	0.0259	0.24	0.8801	ns		
E	0.13	0.01	0.01	6.78	2.54	0.0597	ns		
F	0.53	0.02	0.01	102.61	10.61	0.0005	***		
G	0.74	0.01	0.01	204.78	14.23	0.0001	***		

*Significant at *p* ≤ 0.05, **significant at *p* ≤ 0.01, ***significant at *p* ≤ 0.001, ns: not significant, A: water to plant ratio, B: enzyme amount, C: incubation temperature, D: incubation time, E: shaking speed, F: microwave time, and G: microwave power, Cor total: totals of all information corrected for the mean.

### Optimization of extraction method by BBD

Due to four significant variables including water to plant ratio, enzyme amount, microwave time and power, 29 trial experiments were performed by BBD model. The actual and predicted yields are demonstrated in Table 3. The significance of each variable and the coefficient (R^2^) are also summarized in Table 4. The model equation for the yield of *Z. limonella* essential oil can be found as Y = 6.82 + 0.34A + 0.57B + 0.36F + 0.54G−0.17AF + 0.14AG−0.16BF−0.16BG + 0.18FG–0.26A^2^−0.39B^2^−0.21F^2^−0.38G^2^. Analysis by ANOVA for BBD model demonstrated that the generated model was significant (*p* < 0.0001), and residual lack of fit was not significant (*p* = 0.1971 > 0.05). Adjusted coefficient and correlation coefficient was 0.9711 and 0.9855, respectively.

**Table 3:** Results from BBD model with eight variables.

Run	A	B	F	G	Yield (%)	
					Actual	Predicted
1	12	0.6	40	1500	6.94	6.86
2	10	0.4	20	1500	5.25	5.13
3	10	0.8	30	1700	7.04	7.01
4	8	0.8	30	1500	6.24	6.38
5	10	0.6	30	1500	6.89	6.82
6	12	0.4	30	1500	5.89	5.92
7	8	0.6	30	1700	6.40	6.25
8	8	0.6	30	1300	5.56	5.46
9	10	0.6	40	1700	7.26	7.30
10	10	0.6	30	1500	6.79	6.82
11	12	0.8	30	1500	7.06	7.10
12	10	0.6	20	1300	5.38	5.50
13	10	0.6	30	1500	6.73	6.82
14	12	0.6	20	1500	6.54	6.49
15	12	0.6	30	1300	5.81	5.84
16	10	0.6	30	1500	6.92	6.82
17	10	0.6	20	1700	6.08	6.22
18	12	0.6	30	1700	7.19	7.19
19	10	0.4	40	1500	6.16	6.17
20	10	0.8	30	1300	6.29	6.24
21	8	0.6	20	1500	5.46	5.46
22	8	0.4	30	1500	5.17	5.28
23	8	0.6	40	1500	6.55	6.54
24	10	0.4	30	1700	6.21	6.19
25	10	0.4	30	1300	4.83	4.80
26	10	0.8	40	1500	6.97	6.98
27	10	0.6	30	1500	6.78	6.82
28	12	0.6	40	1300	5.85	5.87
29	12	0.8	20	1500	6.71	6.59

A: water to plant ratio, B: enzyme amount, F: microwave time, and G: microwave power.

**Table 4:** Analysis of variance for the BBD model results.

Source		Sum of squares	Degree of freedom	Mean square	*F* value	*P* value	Inference
Model		12.53	14	0.8949	68.15	< 0.0001	***
*A*		0.17	1	0.17	12.68	0.0031	**
*B*		0.69	1	0.69	52.80	< 0.0001	***
*F*		0.11	1	0.11	7.73	0.0015	**
*G*		0.65	1	0.65	49.34	< 0.0001	***
*AB*		0.0025	1	0.0025	0.19	0.6693	ns
*AF*		0.12	1	0.12	9.06	0.0094	**
*AG*		0.073	1	0.073	5.55	0.0336	*
*BF*		0.11	1	0.11	8.04	0.0132	*
*BG*		0.099	1	0.099	7.56	0.0157	*
*FG*		0.13	1	0.13	9.60	0.0079	**
*A*^*2*^		0.44	1	0.44	33.76	< 0.0001	***
*B*^*2*^		0.96	1	0.96	73.26	< 0.0001	***
*F*^*2*^		0.31	1	0.31	23.76	0.0002	**
*G*^*2*^		0.91	1	0.91	69.53	< 0.0001	***
Residual		0.18	14	0.0131			
Lack of fit		0.16	10	0.0162	2.49	0.1971	ns
Pure Error		0.026	4	0.0064			
Cor total		12.71	28				
S.D.	Mean	C.V.%	Press	R^2^	Adjusted R^2^	Predicted R^2^	Adequate Precision
0.11	6.31	1.82	0.95	0.9855	0.9711	0.9251	30.38

*Significant at *p* ≤ 0.05, **significant at *p* ≤ 0.01, ***significant at *p* ≤ 0.001, ns: not significant, A: water to plant ratio, B: enzyme amount, C: incubation temperature, D: incubation time, E: shaking speed, F: microwave time, and G: microwave power, Cor total: totals of all information corrected for the mean, Cor total: Totals of all information corrected for the mean, S.D.: Standard deviation, C.V: Coefficient of variation.

**Figure 2:**
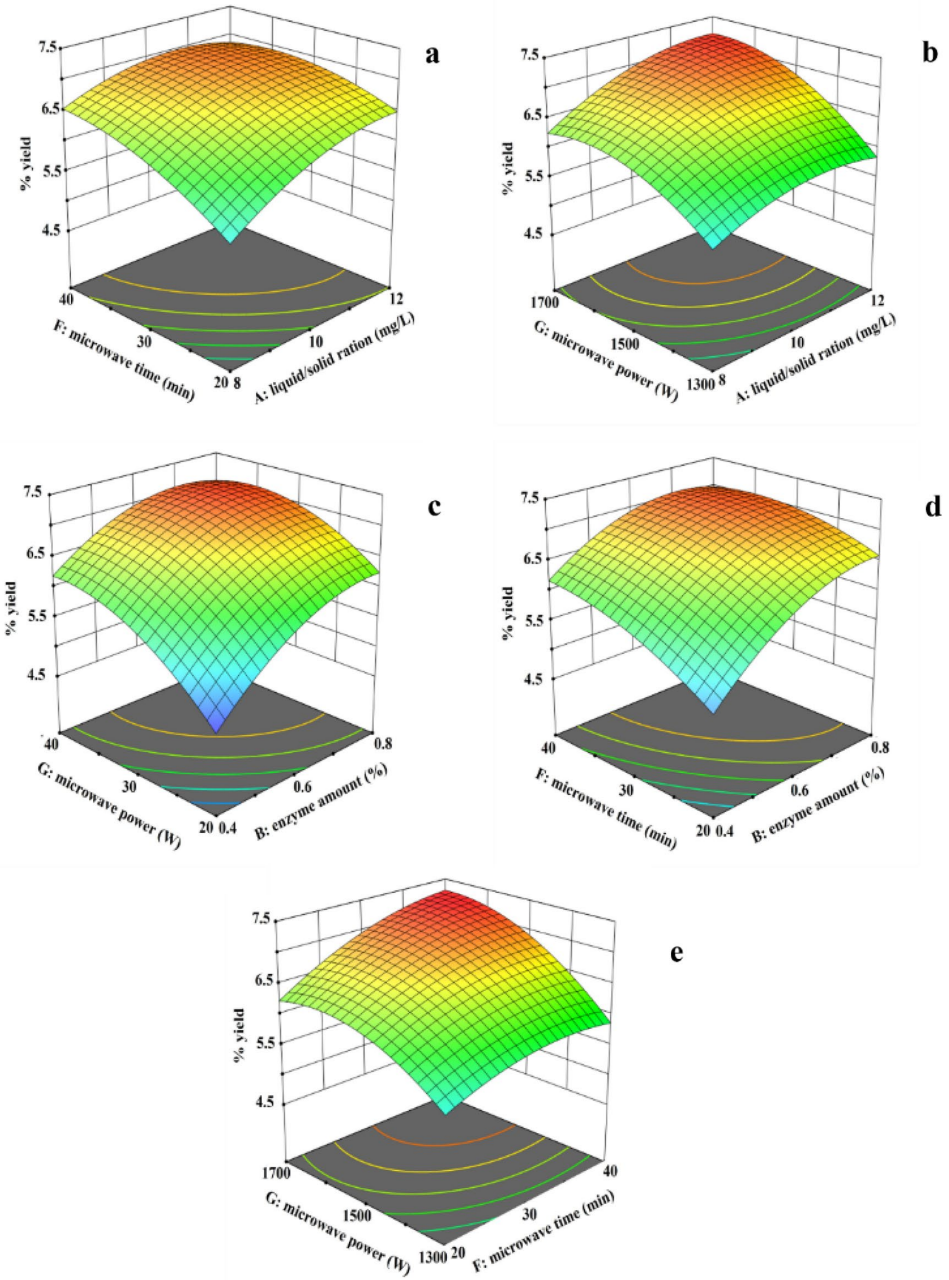
Three-dimensional and contour plots from BBD model; effect between water to plant ratio versus microwave time (**a**); effect between water to plant ratio versus microwave power (**b**); effect between microwave power versus enzyme amount (**c**); effect between microwave power versus microwave time (**d**); and effect between microwave time versus enzyme amount (**e**) on *Z. limonella* essential oil yield.

Three-dimensional and contour plots obtained from BBD results are shown in Fig. 2. These plots demonstrate the effects between two variables on the yield of *Z. limonella* essential oil with the other two independent variables. A weak mutual interaction resulting in a low yield of *Z. limonella* essential oil was found in Figs. 2a, 2c, and 2d which predict the relationship between the water to plant ratio and microwave time, the relationship between microwave power and enzyme amount, and the relationship between microwave power and microwave time, respectively, whereas a strong mutual interaction resulting in a high yield of *Z. limonella* essential oil was found in Fig. 2b, and 2e which illustrate the relationship between the water to plant ratio and microwave power and the relationship between microwave power and microwave time.

**Figure 3:**
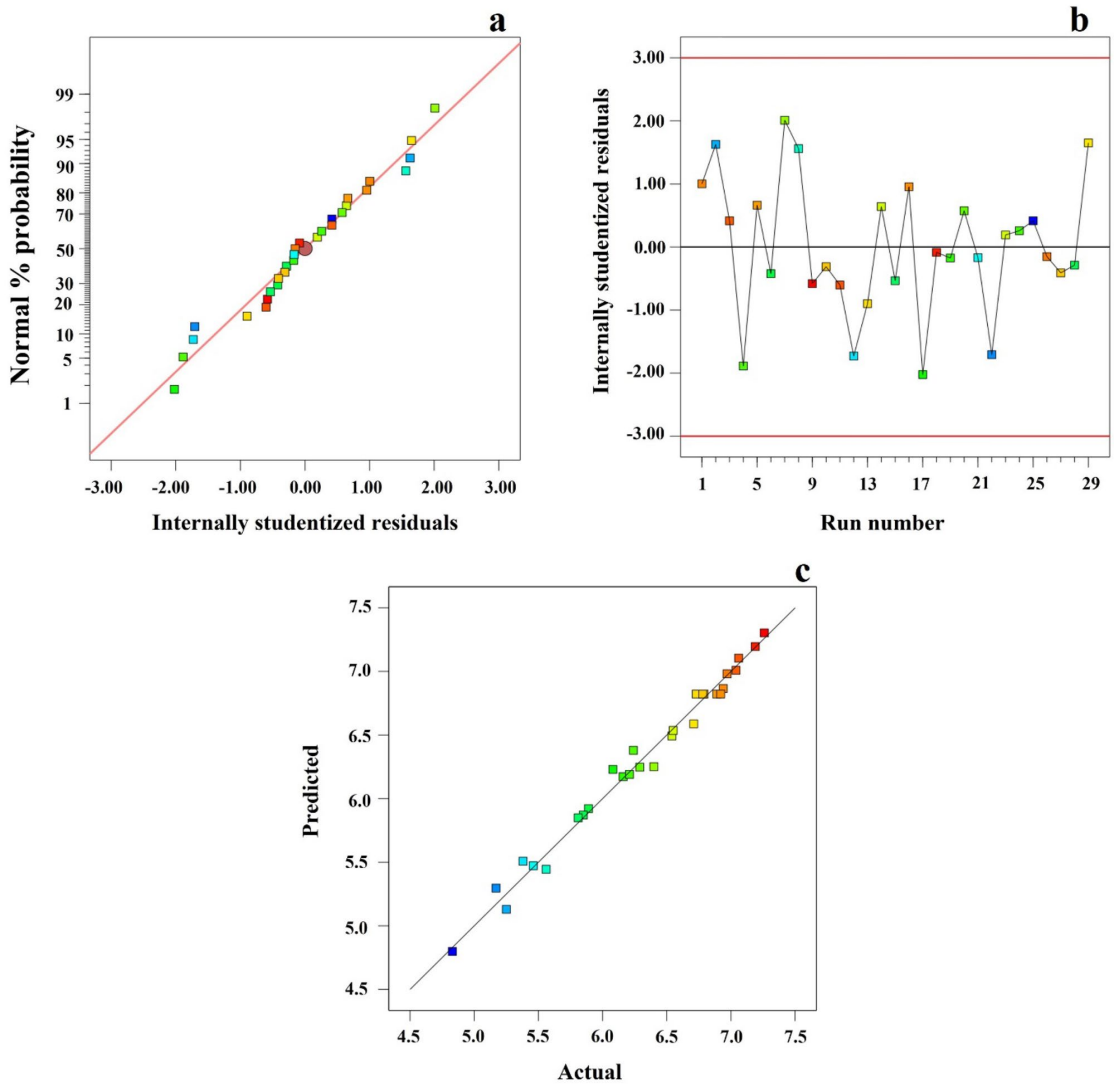
Model adequacy checking obtained by normal plot (**a**) and run number (**b**) versus residuals and predicted versus actual value (**c**).

Checking of model adequacy is illustrated in Fig. 3. Normal plot (Fig. 3a) shows a straight line presenting normal distribution independent on each variable. Residuals versus run number plot (Fig. 3b) shows a random distribution ranging values from + 3 and -3 indicating the quadratic model correlation between the causal factors of the EP-MAE method and the *Z. limonella* oil yield. The plots between predicted versus actual values are shown in Fig. 3c. This plot presents a straight line suggesting that this generated model was accomplished to predict accurately comparing to the actual response values. It was found that proposed model from three residual plots can be applied to optimize extraction method of *Z. limonella* essential oil.

From the BBD experiment, the optimum condition for extracting *Z. limonella* oil by EP-MAE method consisted of a water to plant ratio of 11.16 mL/g, enzyme amount of 0.68%, microwave time of 36.73 min, and power of 1665 W. The predicted yield of *Z. limonella* essential oil was 7.85 mg/g. The values obtained from optimum condition was set in the actual experiments in order to compare yield of *Z. limonella* oil. The results showed that average yield of *Z. limonella* essential oil was 7.89 ± 0.08 mg/g was obtained from the actual experiments. The obtained results were similar to those obtained from the predicted value demonstrating the precision by the generated model. In addition, yield of *Z. limonella* essential oil obtained from EP-MAE was compared to those obtained from MAE and hydrodistillation. As the results, yield of *Z. limonella* essential oil obtained from MAE and hydrodistillation was lower significantly than those found by EP-MAE representing 7.26 ± 0.04 mg/g and 7.04 ± 0.03 mg/g, respectively,

### Analysis of volatile compounds in *Z. limonella* essential oil

The volatile compounds in *Z. limonella* essential oils and their relative peak area obtained from EP-MAE, MAE, and hydrodistillation are listed in Table 5. Similar volatile profile was found among these extraction methods. In total, 51 volatile components were identified with same major compounds of limonene, β-pinene, and α-phellandrene showing percentage ranging between 34.59 and 35.78%, 19.91 and 22.67%, 8.47 and 8.75%, respectively. In addition, concentration of most volatile components obtained by EP-MAE was greater significantly than those obtained by MAE, and hydrodistillation, respectively. These volatile compounds consisted of myrcene, α-terpinene E-β-ocimene, terpinen-4-ol, and germacrene D. However, some compounds such as camphene, iso-isopulegyl acetate, citronellol perilla alcohol, carvacrol possessed similar contents in all extraction methods.

**Table 5:** *Z. limonella* essential oil composition and relative peak areas obtained from EP-MAE, MAE, and hydrodistillation.

No	Compound	Cal RI^a^	Adam RI^b^	EP-MAE	MAE	Hydrodistillation
1	α-Thujene	919	924	14.98	2.17	1.22
2	α-Pinene	927	932	70.89	10.28	4.57
3	Camphene	943	946	0.16	0.03	0.01
4	β-Pinene	971	969	544.75	78.39	29.18
5	trans-meta-Mentha-2,8-diene	975	974	1.72	0.25	0.10
6	Myrcene	985	988	58.44	8.68	3.36
7	α-Phellandrene	999	1002	212.95	30.25	12.42
8	δ-3-Carene	1004	1008	5.80	0.86	0.35
9	α-Terpinene	1011	1014	19.08	1.75	2.21
10	ρ-Cymene	1019	1020	44.78	6.40	2.67
11	Limonene	1025	1024	874.69	122.54	50.69
12	Z-β-Ocimene	1031	1032	28.10	4.11	1.58
13	E-β-Ocimene	1043	1044	144.20	20.95	8.14
14	γ-Terpinene	1054	1054	33.15	3.12	3.52
15	Terpinolene	1084	1086	11.03	1.23	1.04
16	Linalool	1095	1095	26.75	4.26	1.12
17	Nonanal	1099	1099	0.93	0.12	0.07
18	cis-ρ-Menth-2-en-1-ol	1117	1118	10.16	1.29	0.69
19	cis-ρ-Mentha-2,8-dien-1-ol	1130	1133	3.63	0.55	0.19
20	Camphor	1141	1141	0.98	0.16	0.04
21	Sabina ketone	1154	1154	1.51	0.21	0.10
22	Terpinen-4-ol	1175	1175	79.90	16.91	6.76
23	Cryptone	1183	1183	8.04	1.08	0.52
24	α-Terpineol	1188	1186	27.00	3.61	1.46
25	Decanal	1201	1201	11.85	1.81	0.64
26	trans-Piperitol	1204	1207	1.47	0.16	0.11
27	iso-Dihydro carveol	1207	1212	17.44	2.79	0.92
28	trans-Carveol	1216	1215	11.65	1.59	0.66
29	Citronellol	1224	1223	0.87	0.14	0.06
30	cis-Carveol	1227	1226	4.58	0.69	0.25
31	Cumin aldehyde	1237	1238	1.55	0.24	0.09
32	Carvone	1239	1239	9.35	1.30	0.47
33	Decenol	1268	1268	3.55	0.58	0.23
34	p-Menthen-7-al	1272	1273	1.06	0.15	0.24
35	iso-Isopulegyl acetate	1283	1283	1.05	0.16	0.05
36	p-Cymen-7-ol	1288	1289	2.00	0.31	0.20
37	γ-Terpinen-7-al	1290	1290	18.47	2.72	0.99
38	Perilla alcohol	1296	1294	0.52	0.07	0.04
39	Carvacrol	1300	1298	0.69	0.10	0.18
40	Geranyl acetate	1381	1379	19.32	3.27	1.07
41	Z-Isoeugenol	1406	1406	6.71	1.09	0.40
42	E-Caryophyllene	1418	1417	8.76	1.29	0.53
43	α-Humulene	1452	1452	0.91	0.14	0.06
44	Dodecanol	1470	1469	3.04	0.55	0.24
45	Germacrene D	1480	1480	23.84	3.71	1.26
46	δ-Selinene	1493	1492	5.53	0.96	0.41
47	γ-Amorphene	1495	1495	4.65	0.74	0.26
48	Spathulenol	1576	1577	3.25	0.70	0.30
49	Caryophyllene oxide	1582	1582	3.41	0.66	0.24
50	Humulene epoxide II	1607	1608	2.14	0.58	0.16
51	α-Eudesmol	1653	1653	0.39	0.07	0.07

^a^Calculated retention indices on DB5 column.

^b^Retention indices on DB5 column from Adams^25^.

## Discussion

Yield of *Z. limonella* essential oil achieved from cellulase enzymatic pretreatment was significantly higher than those obtained from other methods without pretreatment. The results showed that cellulase enzymatic pretreatment on extraction of *Z. limonella* essential oil was affected on its yield and essential oil composition. The results were similar to those found in previous studies describing that cellulase can hydrolyse the β-1,4 glycosidic bonds in plant cell structure mainly cellulose^8,14,15^. Several results demonstrated the successful of cellulase enzymatic pretreatment in the extraction of essential oil of eaglewood^16^, citrus^17^, basil^18^, cinnamon^8^, and lavender^9^. It was noted that the structure of *Z. limonella* cell walls were broken and depolymerized mainly by cellulase in the pretreatment subsequently releasing intracellular essential oil in the mixture and resulting higher yield in the extraction.

The PBD experiment revealed that four variables including water to plant ratio, enzyme amount, microwave time and power were evaluated as the significant variables affecting on yield and volatile compounds of *Z. limonella* essential oil obtained from the EP-MAE method. Our result was accordance with the study of Liu et al.^8^ reporting these four factors were the major factors on essential oil yield. Karami et al. (2015)^19^ reported that water to plant ratio was one of the important factors in extraction of essential oils. Incomplete extraction may be detected in the extraction using low water to plant ratio whereas complicated separation with undesired products may be found when using high water to plant ratio. Amount of cellulase enzyme was also considered as key factor enhancing the essential oil yield by interaction between substrates and enzyme cell resulting cell wall solubilization^8,20^. However, the efficiency of enzyme was depended on their cost, and extracted sources^21^. In addition, microwave time and power had the most significant impact on yield and volatile compounds of *Z. limonella* essential oil. Prolonged extraction and high power of microwave could lead to the decomposition of volatile compounds, resulting in a lower yield of the *Z. limonella* essential oil^5^. Therefore, these four factors were further used to optimize extraction condition in the EP-MAE method. This optimization aims to achieve cost savings, reduce extraction time, and obtain a high yield along with the essential volatile compounds of *Z. limonella* essential oil.

From BBD results, A high R^2^ value of 0.9855 was found for the response. R^2^ value in this study is acceptable due to higher R^2^ than 0.75. The adjusted and predicted R^2^ value was 0.9711 and 0.9251, respectively. It can be noted that the created model was extremely significant with a *P* value < 0.0001 and an F value was higher than 60. The F and *P* value of the lack of fit revealed nonsignificant difference in variance with a value of 2.49 and 0.1971, respectively. The suggested model also presented adequacy precision of 30.38 which this model could be employed to design variables in the EP-MAE method. As noticed, both the linear and the quadratic terms of the two variables, enzyme amount and microwave power, exhibited significant significance. Additionally, the linear terms from the two variables, water-to-plant ratio and microwave time, demonstrated high significance. The interactive effects between water to plant ratio and microwave time, as well as the effects between enzyme amount and microwave time, and effect between enzyme amount versus microwave time were highly significant while interactive terms of water to plant ratio versus enzyme amount was nonsignificant.

As noticed, both high and low water-to-plant ratios could have an impact on the dissolution, with high ratios potentially leading to incomplete extraction^8,20^. However, use of high microwave power probably destroys the essential oil cells while low microwave power could reduce the dielectric heating^22,23^. Initial radiation in the MAE system could enhance essential oil solubilization while employment of high microwave power may degrade enzymes and plant materials decreasing essential oil yield^5–7^. It was found that the optimal microwave power was 1665 W in this study. As noticed, high power of microwave was not affected on yield of *Z. limonella* essential oil. The optimal conditions obtained by BBD model revealed higher content significantly of all volatile compounds in the EP-MAE than those found in the MAE and hydrodistillation, respectively. The extremely low content of these volatile compounds in the hydrodistillation method may be contributed to the long extraction time resulting completely oxidization, hydrolysis, and even other reactions in the system^22^. The obtained result was similar to the study by Liu et al.^24^ describing that *endo*-borneol content of *Cinnamomum camphor* essential oil was significantly higher by extracting with EP-MAE method comparing to those found by MAE method. This phenomenon may be due to the hydrolysis reaction by enzymolysis pretreatment. In addition, some volatile compounds containing higher dipole force usually demonstrated more drastic response during microwave irradiation resulting more easily separation from the plant material^6,7^.

The EP-MAE method proved successful for the extraction of Z. limonella essential oil. The extraction variables were optimized using RSM with PBD and BBD models. It was found that four variables including water to plant ratio, enzyme amount, microwave time and power were evaluated as significant variables on yield and volatile compounds of *Z. limonella* essential oil. The optimal conditions in EP-MAE were found as follows: water to plant ratio (11.16 mL/g), enzyme amount (0.68%), microwave time (36.73 min), and power (1665 W). The *Z. limonella* essential oil composition and its yield from EP-MAE was compared to those obtained by MAE and hydrodistillation. The optimum conditions of the EP-MAE method led to a significant increase in essential oil yield (7.89 ± 0.08 mg/g) compared to those found by MAE (7.26 ± 0.04 mg/g) and hydrodistillation (7.04 ± 0.03 mg/g). GC–MS analysis revealed an unchanged volatile profile when compared to the profiles obtained using the MAE and hydrodistillation method. The major compounds identified among all methods were limonene, β-pinene, and α-phellandrene. Overall, the EP-MAE improved the yield compared to those obtained by MAE and hydrodistillation, respectively. The EP-MAE holds promise as an extraction technique for *Z. limonella* essential oil without compromising its quality. This suggests that its favorable potential could extend to extracting essential oils from a variety of aromatic plants as well.

## Materials and methods

### Plant material and chemicals

The aerial parts of *Z. limonella* were collected from Pua district, Nan Province, Thailand in September 2021. The collection site access was approved by Mrs. Panid Taewa, the farm owner. The plant material was collected with the consent of the Mae Fah Luang University. No further regulation was required for collection of this plant. In addition, the collection of plant material was complied with the relevant institutional (Mae Fah Luang University), national, and international guidelines and legislation. It was identified by a taxonomist Dr. Jantrararuk Tovaranonte, head of Mae Fah Luang Botanical Garden with a voucher specimen MFU 10064. It was deposited at the Mae Fah Luang Botanical Garden, Mae Fah Luang University, Chiang Rai, Thailand. The *Z. limonella* fruits were dried in oven at 60 °C for 12 h prior kept in plastic bag at room temperature until use. Neutral cellulase (> 10,000 U/g) enzyme was purchased from Sigma-Aldrich Inc. (St. Louis, MO). All analytical grade chemicals from Sigma-Aldrich Inc. (St. Louis, MO) were used in this study.

### Extraction of *Z. limonella* essential oil

The EP-MAE extraction was performed of two parts including enzymatic pretreatment and further extraction in microwave system. This method was applied following method of Liu et al.^8^ Firstly, dried *Z. limonella* fruits were pulverized into a homogeneous size by a disintegrator (50–60 mesh). After that, 100 g of homogeneous *Z. limonella* fruit powder, cellulase enzyme solution at pH 5, and 10 µL of 100 mg/L of 2,6-dimethylpyridine, internal standard, were mixed and further incubated using a digital shaking water bath oscillator (Bioevopeak, Shandong, China) prior subjected to microwave extraction apparatus (ETHOS™, Metrohm, Australia). The excess waters in essential oils were removed using anhydrous Na_2_SO_4_. The obtained *Z. limonella* oils were transferred in sealed amber vials and kept at 4 °C. The MAE method was performed using same optimized extraction conditions described in EP-MAE method without any pretreatment. In addition, *Z. limonella* essential oil was also extracted by hydrodistillation. Briefly, 100 g of *Z. limonella* fruit powder and 1 L of distilled water was placed in flask container. The essential oil was extracted by hydrodistillation with a Clevenger-type apparatus (Apex Chemicals, Thailand) until no essential oil was found. The extraction method in MAE and hydrodistillation was the same as described in EP-MAE. The essential oils obtained by MAE and hydrodistillation were used as control comparing to those obtained by EP-MAE method. The yield was calculated based on dried raw materials.

### Experimental design

PBD model with seven variables was employed to optimize variables influencing the yield of *Z. limonella* essential oil. The method in this study was modified from the study of Liu et al.^8^ In pre-experiments, influences of seven variables were studied to optimize the suitable ranges for extracting of *Z. limonella* essential oil. The mathematical optimization from all variables and the obtained results are shown in Table 1. A first-order polynomial mathematical equation: Y = β0 + ∑βi + Xi where Y is the % yield, β0 and βi instant for the constants of the intercept term and the regression coefficient, respectively. Seven different and independent variables including water to plant ratio, enzyme amount, incubation temperature, incubation time, shaking speed, microwave time, and power were investigated to appraise the comparatively significant variables for extraction of *Z. limonella* essential oil (response). All independent variables are shown in Table 2 designing as − 1 and + 1 for low and high value with twelve experiments. Average *Z. limonella* essential oil yield from each experiment was calculated using Design Expert 13 software.

BBD model was also used to optimize extraction conditions in EP-MAE method with the independent factors in the extraction of *Z. limonella* essential oil. Three levels of each variable were evaluated and represented as low, central, and high, respectively. Twenty-nine experiments were designed using Design Expert 13 software and the results are provided in Table 3. The extraction of *Z. limonella* essential oil was evaluated by the following second-order polynomial equation, and the real yield of essential oil was obtained by the multiregression analysis. Y = β0 + ∑3 i = 1 βiXi + ∑3 i = 1 βiiX2 i + ∑2 i = 1 ∑3 j = i + 1 βijXiXj where Y is the average yield of *Z. limonella* essential oil. β0, βi, βii, and βij are the corresponding regression coefficients of the intercept, linear, quadratic, and interactive terms, respectively; and Xi and Xj are the coded independent variables.

### Identification of volatile compounds by GC–MS

An Agilent 6890 N gas chromatograph connected with electron impact ionization mass-selective detector (Agilent Technologies, Santa Clara, CA, USA). A fused-silica capillary DB5-MS (J&W Scientific, USA) with diameter size of 30 m × 0.25 mm i.d., 0.25 µm was employed to separate volatile compounds. Essential oils obtained by all extraction methods were diluted with hexane with a ratio of 1:100 v/v. Each sample solution (1.0 µL) was injected into the injection port of GC–MS apparatus in split mode (split ratio of 1:50). Helium gas with a rate of 1.0 mL/min was used as a carrier gas. The electron impact ionization mode with the injector, ion source, and interface temperature of 250 °C were used in this work. The programmed temperature was used with initial temperature at 60 °C and then increased to 220 °C at a rate of 3 °C/min. The volatile compounds were identified using MassHunter Acquisition software (Agilent Technologies, Santa Clara, CA, USA) by comparing their mass spectra with those obtained from the Wiley7N and W8N08 and Adams libraries^25^. The retention indices of all volatile compounds were also calculated correlating to those obtained by C_9_-C_16_ n-alkanes. The relative contents of identified compounds were calculated as ratios of their peak areas to the peak areas of the internal standard (2,6- dimethylpyridine).

### Statistical analysis

Design Expert 13 software (Stat-Ease, Minneapolis, USA) was employed in PBD and BBD experiments. Each experiment was performed for five replications. Data were represented as average values. Analysis of variance (ANOVA) was applied to determine the significance of the essential oil yield and volatile profiles among all samples which all data are shown as the mean values ± standard deviation.

## Data Availability

All data generated or analysed during this study are included in this published article.
